# The effects of estrogen on the α2-adrenergic receptor subtypes in rat uterine function in late pregnancy *in vitro*

**DOI:** 10.3325/cmj.2016.57.100

**Published:** 2016-04

**Authors:** Judit Hajagos-Tóth, Judit Bóta, Eszter Ducza, Adrienn Csányi, Zita Tiszai, Anna Borsodi, Reza Samavati, Sándor Benyhe, Róbert Gáspár

**Affiliations:** 1 Department of Pharmacodynamics and Biopharmacy, Faculty of Pharmacy, University of Szeged, Szeged, Hungary; 2 Institute of Biochemistry, Biological Research Centre, Hungarian Academy of Sciences, Szeged, Hungary

## Abstract

**Aim:**

To assess the effect of 17β-estradiol pretreatment on the function and expression of α_2_- adrenergic receptors (ARs) subtypes in late pregnancy in rats.

**Methods:**

Sprague-Dawley SPD rats (n = 37) were treated with 17β-estradiol for 4 days starting from the 18th day of pregnancy. The myometrial expression of the α_2_-AR subtypes was determined by real time polymerase chain reaction and Western blot analysis. *In vitro* contractions were stimulated with (-)-noradrenaline, and its effect was modified with the selective antagonists BRL 44408 (α_2A_), ARC 239 (α_2B/C_), and spiroxatrine (α_2A_). The cyclic adenosine monophosphate (cAMP) accumulation was also measured. The activated G-protein level was investigated by guanosine 5′-O-[gamma-thio]triphosphate (GTPγS) binding assay.

**Results:**

17β-estradiol pretreatment decreased the contractile effect of (-)-noradrenaline via the α_2_-ARs, and abolished the contractile effect via the α_2B_-ARs. All the α_2_-AR subtypes’ mRNA was significantly decreased. 17β-estradiol pretreatment significantly increased the myometrial cAMP level in the presence of BRL 44408 (*P* = 0.001), ARC 239 (*P* = 0.007), and spiroxatrine (*P* = 0.045), but did not modify it in the presence of spiroxatrine + BRL 44408 combination (*P* = 0.073). It also inhibited the G-protein-activating effect of (-)-noradrenaline by 25% in the presence of BRL 44408 + spiroxatrine combination.

**Conclusions:**

The expression of the α_2_-AR subtypes is sensitive to 17β-estradiol, which decreases the contractile response of (-)-noradrenaline via the α_2B_-AR subtype, and might cause changes in G-protein signaling pathway. Estrogen dysregulation may be responsible for preterm labor or uterine inertia via the α_2_-ARs.

In spite of the numerous attempts to explore it, the exact action mechanism and risk of preterm birth still remains one of the biggest challenges in obstetrics and gynecology and a major contributor to perinatal mortality and morbidity, affecting around 9% of births in developed countries (1-4). On the other hand, weak contractions and poor labor outcomes also represent a problem mainly among obese women, increasing the number of cesarean deliveries (5).

Uterine contractility is regulated by several factors, such as the adrenergic system (6) and female sexual hormones (7,8). Progesterone was demonstrated to increase the synthesis of β_2_-ARs during pregnancy (9-11) and the number of activated G-proteins (12,13), which is why it can be combined with β_2_-AR agonists in threatening preterm labor. Myometrial α_1_-AR expression is influenced by female sexual steroid hormones, mainly estrogens. 17β-estradiol decreases the expression of the α_1A_-ARs, but does not influence the expression of α_1D_-ARs (14). However, the effect of estrogens on the myometrial α_2_-AR subtypes is unknown. Considering the fact that estrogens play a major role in myometrial contractions during human parturition (15,16), it is important to know if they have a direct influence on the α_2_-AR subtypes, which are also involved in the mechanism of uterine contractions (17).

The α_2_-ARs have been divided into (18,19) α_2A_, α_2B_, and α_2C_ subtypes. All three receptor subtypes are coupled to the pertussis toxin-sensitive G_i_-protein α-subunit (20) and decrease the activity of adenylyl cyclase (AC) and voltage-gated Ca^2+^ currents, at the same time activating the receptor-operated K^+^ currents (21). The stimulation of these receptors leads to presynaptic feedback inhibition of (-)-noradrenaline release on the adrenergic neurons (18), and mediates a variety of cell functions, such as vasoconstriction, increased blood pressure, and nociception. Furthermore, all three α_2_-AR subtypes were identified in both pregnant and non-pregnant myometrium and were shown to take part in both increased and decreased myometrial contractions (22,23). Under certain circumstances, α_2_-ARs can couple not only to G_i_-proteins but to G_s_-proteins, resulting in the activation of AC (24). On the other hand, pregnancy has been proved to induce a change in the G_i_/G_s_-activating property of the α_2_-ARs in rats, resulting in a differential regulation of myometrial AC activity in mid-pregnancy vs term (25). The α_2B_-ARs were shown to predominate and mediate contraction in last-day-pregnant animals by decreasing the intracellular cAMP level, while α_2A_- and α_2C_-ARs mediate only weak contractions by increasing the cAMP level, which can be regarded as relaxation as they are compared with the effect of (-)-noradrenaline (23).

Since female sexual steroid hormones play an important role in the regulation of the adrenergic receptor system (26), the effect of estrogen on different α_2_-AR subtypes has been investigated. The mRNA expression of the α_2A_-ARs in the spinal cord was increased after estrogen pretreatment (27), which could contribute to the higher prevalence of pain syndromes in women. On the other hand, estrogen was shown to increase the smooth muscle expression of α_2C_-ARs and therefore the cold-induced constriction of cutaneous arteries (28). In addition, it was shown to stimulate the (-)-noradrenaline release in the hypothalamus due to the decreased coupling of the α_2_-adrenoceptors to G protein (29).

Since there are no available data on the effects of 17β-estradiol on the myometrial functions of different α_2_-AR subtypes, the aim of this study was to clarify the changes in expression and function of the α_2A_-, α_2B_-, and α_2C_-AR subtypes after 17β-estradiol pretreatment on the last day of pregnancy in rats by using RT-PCR and Western blot analysis. Since the changes in the intracellular cAMP are crucial in the control of smooth muscle contractions and relaxations, our further aim was to measure the cAMP release after 17β-estradiol pretreatment in the presence of the subtype-specific α_2_-AR antagonists. We also investigated the changes in the G-protein activation of α_2_-ARs using GTPγS binding assay.

## Materials and methods

The animal experimentation was carried out with the approval of the Hungarian Ethics Committee for Animal Research (permission number: IV/198/2013). The animals were treated in accordance with the European Communities Council Directives (86/609/ECC) and the Hungarian Act for the Protection of Animals in Research (XXVIII. tv. 32.§).

### Housing and handling of the animals

Sprague-Dawley rats were obtained from the INNOVO Ltd (Gödöllő, Hungary) and were housed under controlled temperature (20-23°C), in humidity (40%-60%) and light (12 h light/dark regime) regulated rooms. The animals were fed standard rodent pellet diet (INNOVO Ltd, Isaszeg, Hungary), with tap water available *ad libitum*.

### Mating of the animals

Mature female (180-200 g, n = 58) and male (240-260 g, n = 12) Sprague-Dawley rats were mated in a special mating cage with a time-controlled electrically movable metal door separating the rooms for male and female animals. Since rats are usually active at night, the door was opened before dawn. Within 4-5 hours after the possibility of mating, female rats with the presence of copulation plug or a sperm-positive vaginal smear (search was performed under under a microscope at a magnification of 1200 times) were separated. The day of copulation was considered as the first day of pregnancy.

### *In vivo* sexual hormone treatments of the rats

The 17β-estradiol (Sigma Aldrich, Budapest, Hungary) pretreatment of the pregnant animals was started on the day 18 of pregnancy. The compound was dissolved in olive oil. The animals were injected subcutaneously with 5 μg/kg of 17β-estradiol once a day for 4 days (30). On the day 22, the uterine samples were collected and the contractility and molecular pharmacological studies were carried out.

### RT-PCR studies

*Tissue isolation*: Rats (250-300 g) were sacrificed by CO_2_ asphyxiation. Newborn rats were sacrificed by immediate cervical dislocation. The uterine tissues from pregnant animals (n = 5 in each experiment) (tissue between two implantation sites) were rapidly removed and placed in RNAlater Solution (Sigma-Aldrich). The tissues were frozen in liquid nitrogen and stored at -70°C until total RNA extraction.

*Total RNA preparation from tissue*: Total cellular RNA was isolated by extraction with guanidinium thiocyanate-acid-phenol-chloroform according to Chomczynski and Sacchi (31). After precipitation with isopropanol, the RNA was washed with 75% ethanol and then re-suspended in diethyl pyrocarbonate-treated water. RNA purity was controlled at an optical density of 260/280 nm with BioSpec Nano (Shimadzu, Japan); all samples exhibited an absorbance ratio in the range 1.6-2.0. RNA quality and integrity were assessed by agarose gel electrophoresis.

Reverse transcription and amplification of the PCR products was performed by using the TaqMan RNA-to-CTTM 1-Step Kit (Life Technologies, Budapest, Hungary) and the ABI StepOne Real-Time cycler. RT-PCR amplifications were performed as follows: 48°C for 15 min and 95°C for 10 min, followed by 40 cycles at 95°C for 15 sec, and 60°C for 1 min. The generation of specific PCR products was confirmed by melting curve analysis. Table 1 shows the assay IDs for the used primers. The amplification of β-actin served as an internal control. All samples were run in triplicates. The fluorescence intensities of the probes were plotted against PCR cycle numbers. The amplification cycle displaying the first significant increase in the fluorescence signal was defined as the threshold cycle (Ct).

**Table 1:** Assay IDs of the applied primers

TaqMan assays	Assay ID (Life Technologies, Budapest, Hungary)
α_2A_-AR	Rn00562488_s1
α_2B_-AR	Rn00593312_s1
α_2C_-AR	Rn00593341_s1
β-actin	Rn00667869_m1

### Western blot analysis

20 μg of protein per well was subjected to electrophoresis on 4%-12% NuPAGE Bis-Tris Gel in XCell SureLock Mini-Cell Units (Life Technologies) (n = 5 for each α_2_-AR subtype antagonists). Proteins were transferred from gels to nitrocellulose membranes, using the iBlot Gel Transfer System (Life Technologies). The antibody binding was detected with the WesternBreeze Chromogenic Western blot immundetection kit (Life Technologies). The blots were incubated on a shaker with α_2A_-AR, α_2B_-AR, α_2C_-AR, and β-actin polyclonal antibody (Santa Cruz Biotechnology, Santa Cruz, CA, USA, 1:200) in the blocking buffer. Images were captured using the EDAS290 imaging system (Csertex Ltd, Budapest, Hungary), and the optical density of each immunoreactive band was determined with Kodak 1D Images analysis software. Optical densities were calculated as arbitrary units after local area background subtraction.

### Isolated organ studies

The uteri were removed from the 22-day pregnant rats (250-350 g) (n = 8 in each experiment). 5 mm-long muscle rings were sliced from both horns of the uterus and mounted vertically in an organ bath containing 10 mL de Jongh solution (composition: 137 mM NaCl, 3 mM KCl, 1 mM CaCl_2_, 1 mM MgCl_2_, 12 mM NaHCO_3_, 4 mM NaH_2_PO_4_, 6 mM glucose, pH = 7.4). The temperature of the organ bath was maintained at 37°C, and carbogen (95% O_2_ + 5% CO_2_) was perfused through the bath. After mounting, the rings were allowed to equilibrate for approximately 60 min before experiments were started, with a buffer change every 15 min. The initial tension of the preparation was set to about 1.5 g and the tension dropped to about 0.5 g by the end of the equilibration period. The tension of the myometrial rings was measured with a gauge transducer (SG-02; Experimetria Ltd, Budapest, Hungary) and recorded with a SPEL Advanced ISOSYS Data Acquisition System (Experimetria Ltd). In the following step contractions were elicited with (-)-noradrenaline (10^−8^ to 10^-4.5^ M) and cumulative concentration-response curves were constructed in each experiment in the presence of doxazosin (10^−7^ M) and propranolol (10^−5^ M) in order to avoid α_1_-adrenergic and β-adrenergic actions. Selective α_2_-AR subtype antagonists (each 10^−7^ M), propranolol, and doxazosin were left to incubate for 20 minutes before the administration of contracting agents. Following the addition of each concentration of (-)-noradrenaline, recording was performed for 300 s.

### Statistical analysis

Concentration-response curves were fitted and areas under curves (AUC) were evaluated and analyzed statistically with the Prism 4.0 (Graphpad Software Inc. San Diego, CA, USA) computer program. From the AUC values, maximum possible effect (E_max_) and half maximum effective concentration (EC_50_)values were calculated. ANOVA Dunnett test or two-tailed unpaired *t* test were used. *P* < 0.05 was considered as a level of significance.

### Measurement of uterine cAMP accumulation

Uterine cAMP accumulation was measured with a commercial cAMP Enzyme Immunoassay Kit (Cayman Chemical, Ann Arbor, MI, USA). Uterine tissue samples (control and 17β-estradiol treated) from 22-day-pregnant rats (n = 6 in each experiment) were incubated in an organ bath (10 mL) containing de Jongh solution (37°C, perfused with carbogen). Isobuthylmethylxantine (10^−3^ M), doxazosin (10^−7^ M), propranolol (10^−5^ M) and the investigated subtype-selective α_2_-AR antagonists (each 10^−7^ M) were incubated with the tissues for 20 minutes, and (-)-noradrenaline (3 × 10^−6^ M) were added to the bath for 10 minutes. At the end of (-)-noradrenaline incubation period, forskolin (10^−5 ^M) was added for another 10 min. After stimulation, the samples were immediately frozen in liquid nitrogen and stored until the cAMP extraction (32). Frozen tissue samples were then ground, weighed, homogenized in 10 volumes of ice-cold 5% trichloroacetic acid and centrifuged at 1000g for 10 min. The supernatants were extracted with 3 volumes of water-saturated diethyl ether. After drying, the extracts were stored at -70°C until cAMP assay. Tissue cAMP levels were expressed in pmol/mg tissue.

### GTPγS binding assay

The uteri were removed (n = 5 in each experiment) and homogenized in 20 volumes (w/v) of ice-cold buffer (10 mM Tris-HCl, 1 mM EDTA, 0.6 mM MgCl_2_, and 0.25 M sucrose, pH 7.4) with an Ultra Turret T25 (Janke & Kunkel, Staufen, Germany) homogenizer, and the suspension was then filtered on four layers of gauze and centrifuged (40,000g, 4°C, 20 min). After centrifugation, the pellet was resuspended in a 5-fold volume of buffer. The protein contents of the samples were diluted to 10 mg protein/sample. Membrane fractions were incubated in a final volume of 1 mL at 30°C for 60 min in Tris-EGTA buffer (pH 7.4) composed of 50 mM Tris-HCl, 1 mM EGTA, 3 mM MgCl_2_, 100 mM NaCl, containing 20 MBq/0.05 cm^3^ [^35^S]GTPγS (0.05 nM) (Sigma Aldrich) together with increasing concentrations (10^−9^-10^−5^ M) of (-)-noradrenaline. BRL 44408, ARC 239, and spiroxatrine were used in a fixed concentration of 0.1 μM. For the blocking of α_1_- and β-ARs, doxazosin and propranolol were used in a fixed concentration of 10 μM. The determination of total and non-specific binding, filtration, washing procedure, and radioactivity detection were performed (33). The [^35^S]GTPγS binding experiments were performed in triplicate and repeated at least three times. G_i_ protein was inhibited with pertussis toxin (Sigma Aldrich) at a concentration of 500 ng/mL after the addition of protein and GDP to the Tris-EGTA buffer 30 min before [^35^S]GTPγS.

## Results

### RT-PCR and Western blot studies

**Figure 1:**
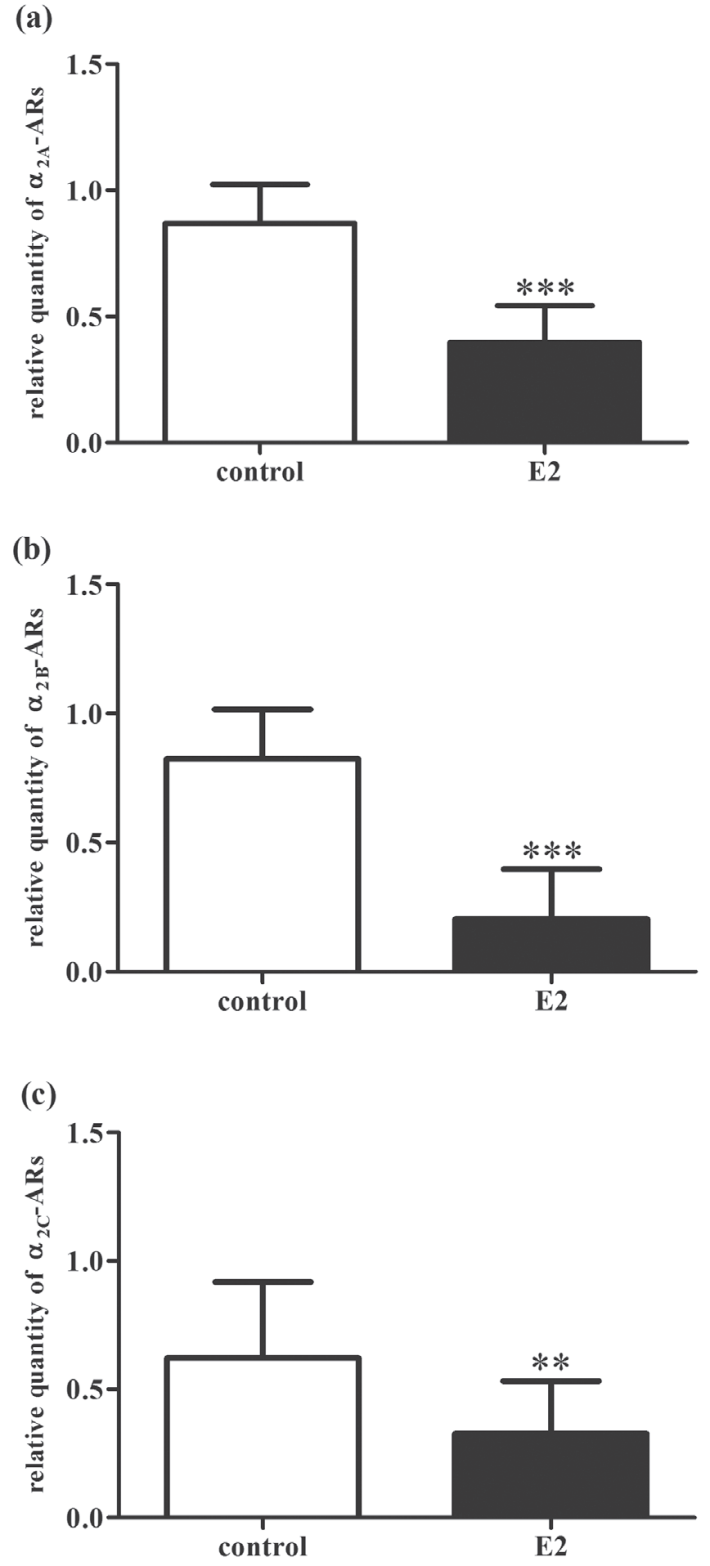
Changes in the myometrial mRNA expression of the α_2A_- (**A**), α_2B_- (**B**), and α_2C_- adrenergic receptors (ARs) (**C**) after 17β-estradiol pretreatment (n = 5). The statistical analyses were carried out with a two-tailed unpaired *t* test. **P = 0.005; ***P < 0.001.

**Figure 2:**
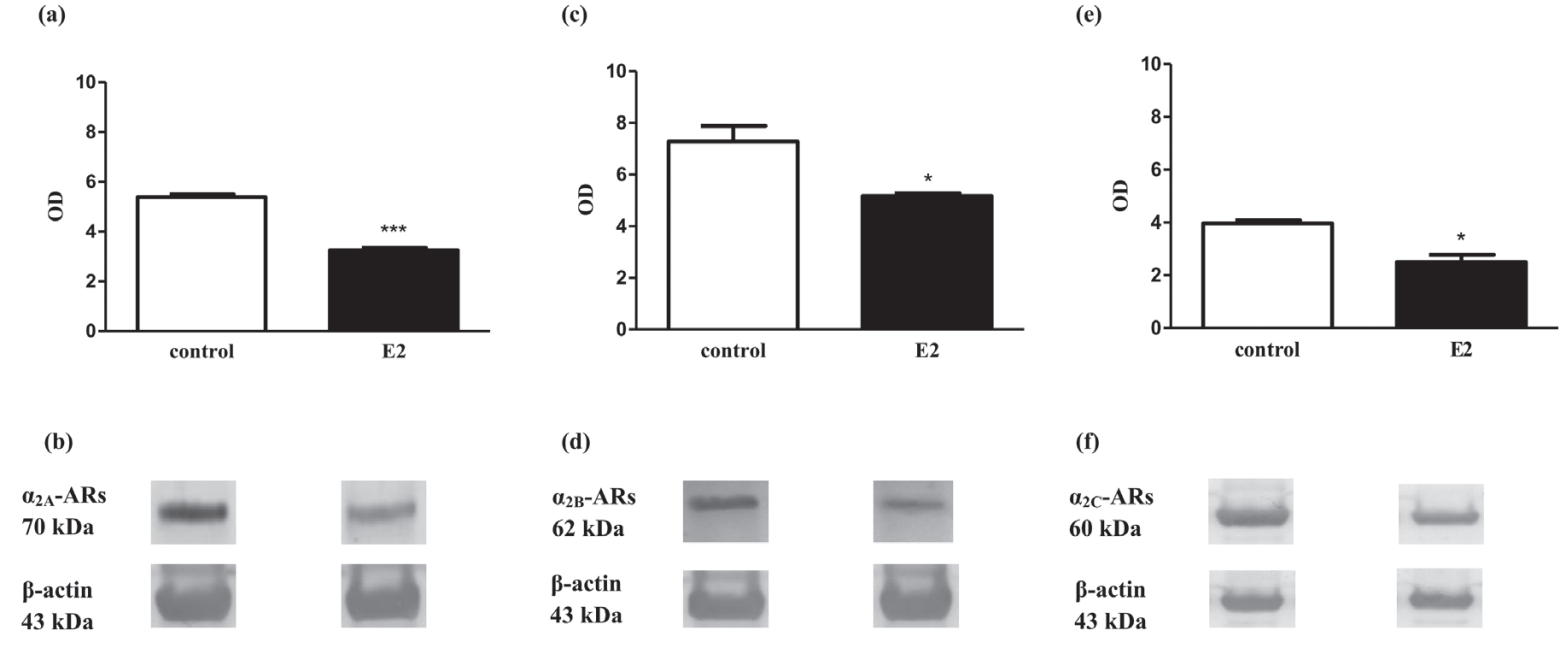
Changes in the α_2_- adrenergic receptor (AR) levels in the 22-day pregnant rat myometrium after 17β-estradiol pretreatment (n = 5). The α_2_-AR and β-actin Western blot products for α_2A_- (**B**), α_2B_- (**D**). and α_2C_-ARs (**F**). The 70, 62, and 60 kDa proteins relate to α_2AR_-, α_2B_-, and α_2C_-ARs and β-actin, respectively. The antibody binding was expressed as optical density (OD) data (**A)** for α_2AR,_ (**C)** for α_2B,_ and (**E)** for α_2C_-ARs. The y-axis shows the ratio of α_2_-AR/ β-actin protein optical density. The statistical analyses were carried out with a two-tailed unpaired *t* test. * *P* < 0.027; *** *P* < 0.001

The mRNA expression of all α_2_-AR subtypes (Figure 1A, 1C, 1E) was significantly decreased (*P* < 0.048) after 17β-estradiol pretreatment compared to non-treated uteri (*P* < 0.001). Western blot analysis at the level of protein expression revealed significant decrease (*P* < 0.027) in each α_2_-AR subtype, corresponding to the PCR results (Figure 2A-F).

### Isolated organ studies

**Figure 3:**
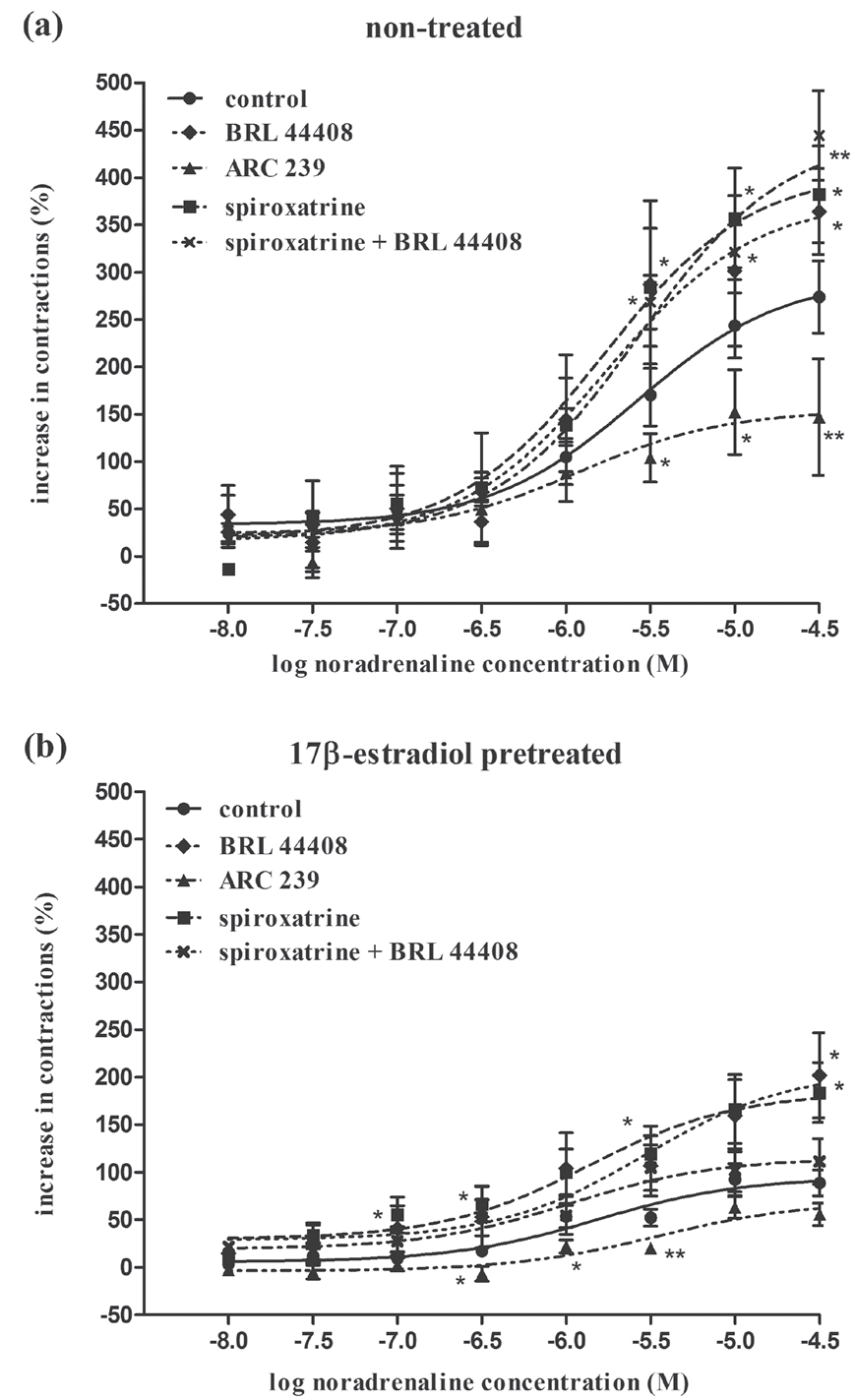
Effects of the subtype-selective α_2A_-adrenoceptor antagonist BRL 44408, the α_2B/C_-adrenoceptor antagonist ARC 239, and the α_C_-adrenoceptor antagonist, spiroxatrine on the (-)-noradrenaline-evoked contractions in the 22-day-pregnant rat myometrium (**A**) and after 17β-estradiol pretreatment (**B**) (n = 8). The studies were carried out in the presence of the β-adrenoceptor antagonist, propranolol (10^−5^ M) and the α_1_-adrenoceptor antagonist, doxazosin (10^−7^ M) in each case. The change in contraction was calculated using the area under the curve and expressed in % ± SEM. The statistical analyses were carried out with the ANOVA Dunnett test. *P < 0.05; **P < 0.01; ***P < 0.001.

In the 22-day-pregnant myometrium, (-)-noradrenaline in the concentration range of 10^−8^ to 10^-4.5^ M increased (*P* = 0.001) myometrial contractions (Figure 3A). After 17β-estradiol pretreatment, the myometrial contracting effect of (-)-noradrenaline was decreased (*P* = 0.005). The EC_50_ and E_max_ values of the curves are shown in Table 2.

**Table 2:** Changes in the uterus-contracting effect of (-)-noradrenaline (EC_50_ and E_max_ values) in the absence of α_2_-antagonists, or in the presence of an α_2A_-antagonist, an α_2B/C_-antagonist, an α_2C_-antagonist, or α_2A_-antagonist and α_2C_-antagonist in the 22-day-pregnant rat after 17β-estradiol pretreatment (n = 8 in each experiment)

	EC_50_ (M ± SD)	E_max_ (% ± SD)
Control		
non-treated	2.6×10^-6^ ± 6.6×10^-6^	274.1 ± 57.8
17β-estradiol pretreated	1.5×10^-6^ ± 1.8×10^-5 ns^	88.7 ± 35.5 ^**^
BRL 44408		
non-treated	1.8×10^-6^ ± 1.6×10^-5^	364.3 ± 83.4
17β-estradiol pretreated	2.9×10^-6^ ± 7.1 ×10^- 6 ns^	202.0 ± 59.9 ^*^
ARC 239		
non-treated	1.2×10^-6^ ± 2.9×10^-6^	147.1 ± 82.0
17β-estradiol pretreated	3.5×10^-6^ ± 7.8×10^-5 ns^	55.9 ± 36.71^*^
Spiroxatrine		
non-treated	1.6×10^-6^ ± 1.2×10^-5^	382.4 ± 103.5
17β-estradiol pretreated	1.4×10^-6^ ± 1.5×10^-6 ns^	183.7 ± 53.6 ^*^
Spiroxatrine + BRL 44408		
non-treated	2.9×10^-6^ ± 1.9×10^-6^	444.6 ± 79.7
17β-estradiol pretreated	1.1×10^-6^ ± 4.6 ×10^- 6 ns^	111.4 ± 59.0 ^***^

EC_50_ – the concentration of (-)-noradrenaline alone or in the presence of an α_2_-AR antagonist which elicits half of the maximum contracting effect of (-)-noradrenaline. E_max_ – the maximum contracting effect of (-)-noradrenaline alone or in the presence of an α_2_-AR antagonist. ns – not significant. *P < 0.05; **P < 0.01; ***P < 0.001. Significance levels were calculated in comparison with non-treated values.

In the presence of the α_2A_-AR antagonist BRL 44408, 17β-estradiol pretreatment increased the (-)-noradrenaline evoked contractions compared to the 17β-estradiol-treated control (*P* = 0.004) (Figure 3B). However, it decreased (*P* = 0.029) the myometrial contracting effect of (-)-noradrenaline compared to the BRL 44408-treated control (Table 2).

In the presence of the α_2B/C_-AR antagonist ARC 239, 17β-estradiol pretreatment decreased the myometrial contractions compared to the 17β-estradiol-treated control (*P* = 0.007) (Figure 3B) and decreased it (*P* = 0.045) compared to the ARC 239-treated control (Table 2).

In the presence of spiroxatrine, 17β-estradiol increased the maximum contracting effect of (-)-noradrenaline compared to the 17β-estradiol-treated control (*P* < 0.001) (Figure 3B), but decreased it (*P* = 0.003) compared to the spiroxatrine-treated control (Table 2).

In the presence of the combination of BRL 44408 and spiroxatrine, 17β-estradiol did not change the maximum myometrial contracting effect of (-)-noradrenaline compared to the 17β-estradiol-treated control (Figure 3B), but decreased it (*P* < 0.001) compared to the BRL 44408+spiroxatrine treated control (Table 2).

### cAMP studies

**Figure 4:**
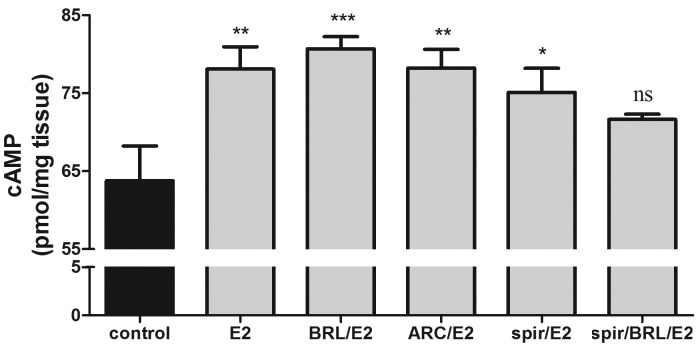
Effects of the subtype-selective α_2A_-adrenoceptor antagonist, BRL 44408, the α_2B/C_-adrenoceptor antagonist, ARC 239, and the α_2C_-adrenoceptor antagonist, spiroxatrine on the myometrial cAMP level (pmol/mg tissue ± standard deviation) in the presence of 3-isobutyl-1-methylxanthine (IBMX) (10^−3^ M) and forskolin (10^−5^ M) (control) in the 22-day-pregnant rat (n = 6) after 17β-estradiol pretreatment. The statistical analyses were carried out with ANOVA followed by Dunnett's Multiple Comparison Test. *P = 0.046, **P < 0.007, ***P = 0.001.

17β-estradiol pretreatment increased the myometrial cAMP level (*P* = 0.007) (Figure 4) produced in the presence of (-)-noradrenaline. 17β-estradiol pretreatment also increased the myometrial cAMP level in the presence of (-)-noradrenaline and BRL 44408 (*P* = 0.001), ARC 239 (*P* = 0.007), and spiroxatrine (*P* = 0.045). However, it did not change the cAMP level in the presence of the spiroxatrine + BRL 44408 combination.

### [^35^S]-GTPγS binding assay studies

**Figure 5:**
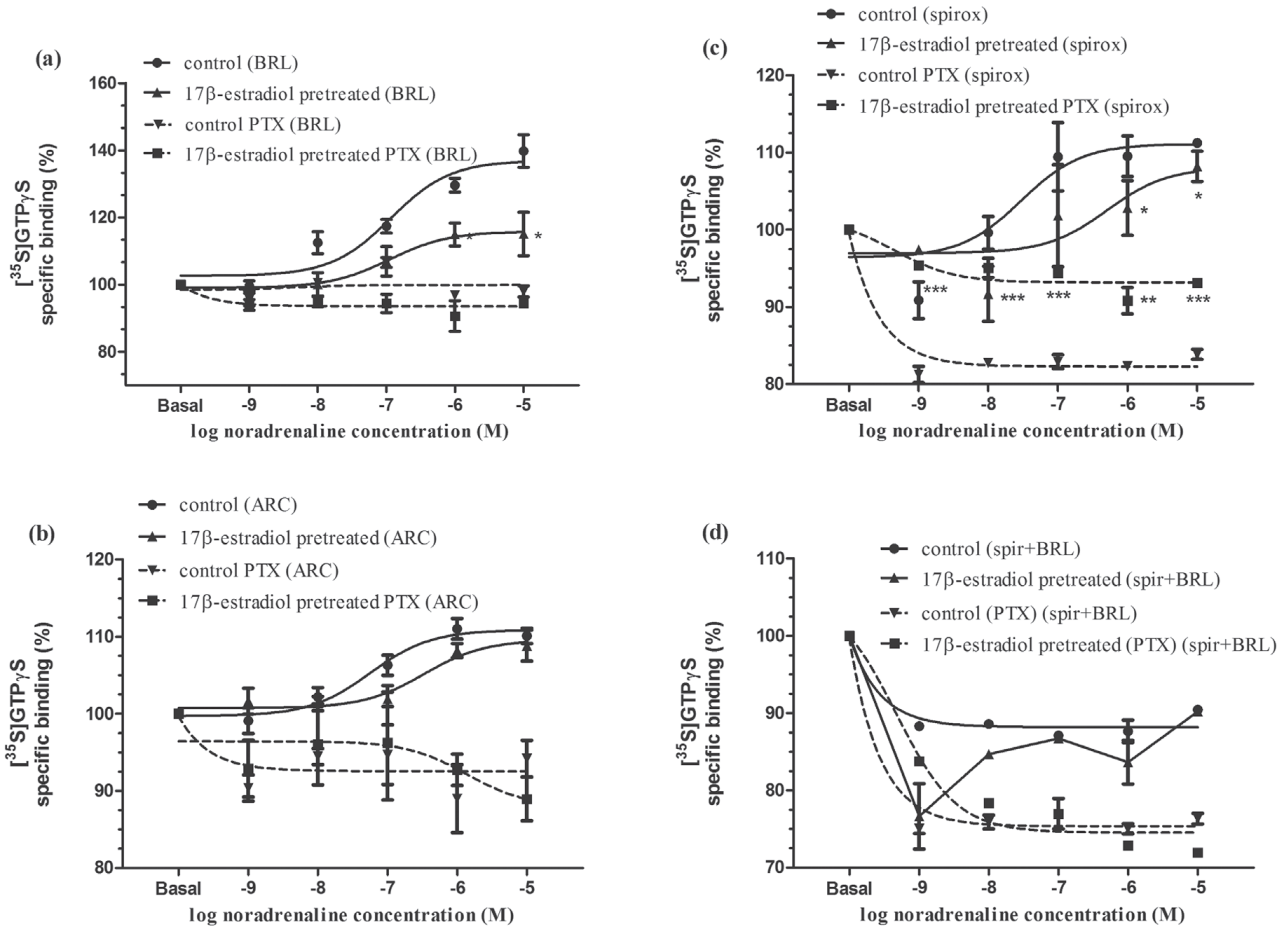
Changes induced by various concentrations of noradrenaline in [^35^S]GTPγS binding in the presence of subtype-selective α_2A_-antagonist BRL 44408 (**A**), the α_2B/C_- antagonist ARC 239 (**B**), the α_2C_- antagonist spiroxatrine (**C**), and the BRL 44408-spiroxatrine combination (**D**) following pretreatment with 17β-estradiol (n = 5). In all cases, the β-adrenoceptors and α_1_-adrenoceptors were inhibited by propranolol and doxazosin. Basal refers to the level of [^35^S]GTPγS binding without substance. The statistical analyses were carried out with a two-tailed unpaired *t* test. *P < 0.038; *P < 0.004, ***P < 0.001.

In the presence of BRL 44408, (-)-noradrenaline increased the [35S]GTPγS binding, which was significantly decreased after 17β-estradiol pretreatment (*P* = 0.038). In the presence of pertussis toxin, the [35S]GTPγS binding-stimulating effect of (-)-noradrenaline ceased, and 17β-estradiol pretreatment did not modify this effect (Figure 5A).

In the presence of ARC 239, (-)-noradrenaline increased (*P* < 0.001) the [35S]GTPγS binding similarly to 17β-estradiol pretreatment. In the presence of pertussis toxin, (-)-noradrenaline slightly decreased the [35S]GTPγS binding, which was not changed after 17β-estradiol pretreatment (Figure 5B).

In the presence of spiroxatrine, (-)-noradrenaline increased the [35S]GTPγS binding (*P* < 0.001), which was slightly decreased (*P* = 0.037) after 17β-estradiol pretreatment. In the presence of pertussis toxin, however, (-)-noradrenaline decreased the [35S]GTPγS binding below the basal level from a concentration of 1 × 10^−9^ M (*P* < 0.001). In the presence of pertussis toxin, 17β-estradiol pretreatment abolished the [^35^S]GTPγS binding-inhibitory effect of (-)-noradrenaline (Figure 5C).

In the presence of spiroxatrine+BRL 44408 combination, (-)-noradrenaline inhibited the [^35^S]GTPγS binding, and 17β-estradiol further inhibited the [^35^S]GTPγS binding of (-)-noradrenaline and abolished the dose-dependency of noradrenalin action. In the presence of pertussis toxin, the spiroxatrine+BRL 44408 combination dose-dependently inhibited the [^35^S]GTPγS binding of (-)-noradrenaline, similarly to 17β-estradiol pretreatment (Figure 5D).

## Discussion

Since estrogens and the adrenergic system play a major role in myometrial contractions during human gestation, the main focus of our study was to clarify the effects of estrogen on the α_2_-AR subtypes in late pregnant uterine function *in vitro*. The estrogen- α_2_-AR connection was investigated via the effects of subtype-selective antagonists after 17β-estradiol pretreatment on the (-)-noradrenaline-stimulated contractions. The experiments were carried out in the presence of the α_1_-AR blocker doxazosin and the β-AR blocker propranolol in order to avoid α_1_- or β-adrenergic actions.

17β-estradiol pretreatment decreased the mRNA and protein expression of the myometrial α_2_-AR subtypes and (-)-noradrenaline-evoked myometrial contraction via the α_2_-ARs, which is similar to our earlier findings with α_1A_-ARs (14). According to these findings, we can claim that estrogen differently affects the expression of the α_2_-ARs in various tissues, as it increases the expression of the receptors in the spinal cord and cutaneous arteries (27,28).

In isolated organ bath studies, 17β-estradiol pretreatment decreased (-)-noradrenaline-evoked myometrial contractions via the α_2_-ARs, although it did not modify the myometrial relaxing effect via the α_2A_-ARs. However, it abolished the myometrial contraction-increasing effect via the α_2B_-ARs. Since there are no available antagonists to produce only α_2C_-AR stimulation (ie, α_2A/B_-AR blockers), we can only presume that 17β-estradiol did not modify the myometrial relaxing effect via the α_2C_-ARs.

To explain why weaker myometrial contractions via the α_2B_-AR subtype occurred after 17β-estradiol pretreatment, we measured the myometrial cAMP level, as the changes in the cAMP level are involved in the myometrial effect of the α_2_-ARs. 17β-estradiol pretreatment increased the myometrial cAMP level, which also proves the decreased myometrial contracting effect of (-)-noradrenaline via the α_2_-ARs. It did not modify the cAMP level via the α_2A_-ARs, which is in accordance with our previous study (23). However, it increased the myometrial cAMP level via the α_2B_-ARs, which can explain the weaker myometrium contracting effect of (-)-noradrenaline.

The α_2_-ARs can couple not only to the G_i_ protein α-subunit, but under certain circumstances, also to G_s_ proteins (24). Estrogen was also shown to decrease the coupling of the α_2_-adrenoceptors to G protein (29). To find an explanation for the cAMP changes, we measured the myometrial [^35^S]GTPγS binding of the α_2_-AR subtypes after 17β-estradiol pretreatment and in the presence of pertussis toxin, whose inhibitory action is specific for the G_i_ protein. In the presence of pertussis toxin, 17β-estradiol did not modify the [^35^S]GTPγS binding of the α_2A_-ARs, but it reversed the effect of (-)-noradrenaline on [^35^S]GTPγS binding via the α_2A_- and α_2B_-ARs (with spiroxatrine). These findings show that 17β-estradiol modifies the coupling of the α_2B_-ARs, but does not change the G protein binding of the α_2A_-ARs. To prove this hypothesis, we measured the myometrial [^35^S]GTPγS binding of the α_2B_-AR subtype in the presence of spiroxatrine+BRL 44408. 17β-estradiol decreased the amount of activated G-protein, which is probably a consequence of 17β-estradiol-induced uncoupling of α_2B_-ARs from the G proteins (29). This process did not change myometrial contraction as compared with the hormone-treated control.

In the light of our results, we conclude that the functions of the α_2_-AR subtypes are influenced by the female sexual steroid, 17β-estradiol. It decreases the expressions of the α_2_-AR subtypes and increases uterine cAMP level. It does not modify the myometrial relaxing effect via the α_2A_- and α_2C_-ARs. In case of these receptors we suppose that the 17β-estradiol treatment mainly induces the activation of βγ subunit of G_i_ protein, increasing the smooth muscle cAMP level (17). In case of α_2B_-ARs, 17β-estradiol alters the myometrial contracting effect of (-)-noradrenaline by reduced coupling of the receptor to G_i_ protein.

A limitation of our study is that we did not carry out any studies on human myometrium, and there might be differences in the function of the rat and human myometrial α_2_-AR subtypes. However, our present findings give a better understanding on the complex physiology of changes during pregnancy, as estrogen is the predominantly expressed hormone during human parturition at term (15,34), which, together with the α_2_-ARs, plays an essential role in myometrial contractility. It was also demonstrated that estrogen level in the amniotic fluid was elevated in uterine inertia (35), which might be caused by the decreased myometrial contractility via the α_2_-AR subtypes. Therefore, estrogen level dysregulation during pregnancy might change the function of the α2-AR subtypes and result in either preterm labor or labor delay. We would like to extend these preclinical studies for premature birth models in rats. We suppose that either subtype-specific agonists or antagonists can be used as a target for drugs against abnormal myometrial contractility.
